# Book Review: *The Magic Cape*: A Volunteer Storytelling Experience Among School-Aged Children

**DOI:** 10.5334/cie.29

**Published:** 2021-01-28

**Authors:** Suzana Cascao

**Affiliations:** 1 University of Luxembourg, LU

**Keywords:** hospitalised children, resilience, school-aged children, workshops, storytelling, volunteers

## Abstract

*The Magic Cape* is a story born out of the initiative of the author, a primary school teacher, and other volunteers from the non-profit Compagnia del Mantello organization. The book tells the story of a wizard who discovers he has the power to transform the world into a better place. As such, together with his friends, he founds Chocolate Town. The book has become part of a two-pronged project involving mainstream students and children who are hospitalized, consisting of a series of interactive and communication activities, as follows: Mainstream children read the book using the materials provided, then the teacher invites the children to create their own stories. Finally, the stories are presented by volunteers to children in hospitals.

*The Magic Cape* is a story born out of the experiences of the author, a primary school teacher, and other volunteers from the non-profit Compagnia del Mantello organization, whose work focuses on creating support paths for hospitalized children. The book tells the story of a wizard who discovers he has the power to transform the world into a better place and, together with his friends, founds Chocolate Town. He is always happy, optimistic, and never gives up. He loves the sea and the outdoors, and is gifted with an uncommon poetic sensitivity. One day he realizes he has lost his hat. In order to find it, the wizard decides to embark on a trip that will allow him to meet old and new friends. By closing his thumb and index finger together, he mimics a monocle and creates a round hole that brings him closer to the sky and the stars.

From the very beginning, *The Magic Cape* conveys the message that thoughts and actions can change us and the reality in which we live. What is important is not to give up in the face of difficulties but to remain positive and optimistic. Other educational cues can be found in the capacity the wizard has of listening to himself and others, combined with an inquisitive outlook on the world. Moreover, the narrative highlights the importance of welcoming “the other” without prejudice.

Compagnia del Mantello originally promoted the book and the initiatives related to it in pediatric hospitals, with volunteer actors presenting the wizard’s adventures to the children using costumes and props to capture their attention. After the performance, the children are involved in a creative workshop related to the story. At the end of the workshop, each patient receives a copy of *The Magic Cape* as a memento.

Born from the experience in hospitals, the project was expanded to schools where it involves a series of interactive and communication activities, as follows: mainstream children first read the book using the materials provided and then the teacher invites them to create their own stories. These new stories are subsequently presented by the volunteers to hospitalized children in the usual way, collated into new books to give to the hospitalized children. In turn, the performances of actors in the hospitals are made available to schoolchildren through YouTube.

To date, 11,000 copies of *The Magic Cape* have been donated to pediatric hospitals and schools. Hospital workshops have been proposed and staged in a dozen hospitals throughout Italy.

The schools involved in the project include 13 Italian institutes and one school in the United States.

The book’s tales symbolically portray many of the realities experienced by children. Each story is open-ended, allowing the readers to escape and complete any of the characters’ looks as well as landscapes according to their own preference and imagination. In fact, the book departs from the classic storytelling typical of children’s literature, both when read and re-written at school and when performed in hospital. There is no real hero, no co-principal character nor an antagonist; there is no dramatic tension nor the usual twist in the plot to be solved. In fact, It remains unknown whether the wizard’s quest for his hat will have a positive outcome because it is a story with an open ending. Further, the book has none of the creepy fairy tales and disturbing details or morale, typically found in children’s stories. It is an open-ended story that each child can continue “composing.” That is, there is no conclusion; on the contrary, there is a protagonist who serves as a connector, a series of proactive and cooperative helpers, as well as moments of exploration that stimulate different paths and perspectives, all full of possibilities, towards an open conclusion. It is the open conclusion that activates the imagination of the individual reader, stimulating her/him to create new stories and new illustrations; for example, by proposing new tools and instruments that the wizard might need.

The book’s structure encourages the capacity to be present here and now by integrating past and future emotions in a functional way. With their creative narratives, children feel the positive touch of what they are bringing to their peers who are hospitalized. Finally, it encourages its readers to express gratitude, reflect on their emotions, and think about their actions, ultimately leading to better self-esteem and increased resilience.

***The Magic Cape (Mago Mantello)*, by Francesco Domenico Giannino. Compagnia del Mantello ODV Rome 2018.**

*The Magic Cape* is available in English, Spanish, Russian, Farsi, Slovak and Polish. It is available at https://www.compagniadelmantello.it/index.php/sostienici/regalamagomantello or info@compagniadelmantello.it.

